# Implications of response shift for micro-, meso-, and macro-level healthcare decision-making using results of patient-reported outcome measures

**DOI:** 10.1007/s11136-021-02766-9

**Published:** 2021-03-02

**Authors:** Richard Sawatzky, Jae-Yung Kwon, Ruth Barclay, Cynthia Chauhan, Lori Frank, Wilbert B. van den Hout, Lene Kongsgaard Nielsen, Sandra Nolte, Mirjam A. G. Sprangers, the Response Shift – in Sync Working Group

**Affiliations:** 1 School of Nursing, Trinity Western University, 7600 Glover Road, Langley, BC V2Y 1Y1 Canada; 2 Centre for Health Evaluation and Outcome Sciences, Providence Health Care Research Institute, Vancouver, Canada; 3 Sahlgrenska Academy, University of Gothenburg, Gothenburg, Sweden; 4 School of Nursing, University of British Columbia, Vancouver, Canada; 5 School of Nursing, University of Victoria, Victoria, Canada; 6 Department of Physical Therapy, College of Rehabilitation Sciences, Rady Faculty of Health Sciences, University of Manitoba, Winnipeg, Canada; 7 Patient Representative, Wichita, KS USA; 8 Behavioral & Policy Sciences, RAND Corporation, Arlington, VA USA; 9 Medical Decision Making, Department of Biomedical Data Sciences, Leiden University Medical Center, Leiden, The Netherlands; 10 Department of Haematology, Quality of Life Research Center, Odense University Hospital, Odense, Denmark; 11 Department of Internal Medicine and Cardiology, Regional Hospital Viborg, Viborg, Denmark; 12 ICON GmbH, Munich, Germany; 13 Corporate Member of Freie Universität Berlin, Humboldt-Universität Zu Berlin, and Berlin Institute of Health, Medical Department, Division of Psychosomatic Medicine, Charité – Universitätsmedizin Berlin, Berlin, Germany; 14 Department of Medical Psychology, Amsterdam University Medical Centers, Research Institute Amsterdam Public Health, Amsterdam, The Netherlands

**Keywords:** Patient-reported outcomes, Response shift, Healthcare decision-making, Patient, Organization, Health policy

## Abstract

**Purpose:**

Results of patient-reported outcome measures (PROMs) are increasingly used to inform healthcare decision-making. Research has shown that response shift can impact PROM results. As part of an international collaboration, our goal is to provide a framework regarding the implications of response shift at the level of patient care (micro), healthcare institute (meso), and healthcare policy (macro).

**Methods:**

Empirical evidence of response shift that can influence patients’ self-reported health and preferences provided the foundation for development of the framework. Measurement validity theory, hermeneutic philosophy, and micro-, meso-, and macro-level healthcare decision-making informed our theoretical analysis.

**Results:**

At the micro-level, patients’ self-reported health needs to be interpreted via dialogue with the clinician to avoid misinterpretation of PROM data due to response shift. It is also important to consider the potential impact of response shift on study results, when these are used to support decisions. At the meso-level, individual-level data should be examined for response shift before aggregating PROM data for decision-making related to quality improvement, performance monitoring, and accreditation. At the macro-level, critical reflection on the conceptualization of health is required to know whether response shift needs to be controlled for when PROM data are used to inform healthcare coverage.

**Conclusion:**

Given empirical evidence of response shift, there is a critical need for guidelines and knowledge translation to avoid potential misinterpretations of PROM results and consequential biases in decision-making. Our framework with guiding questions provides a structure for developing strategies to address potential impacts of response shift at micro-, meso-, and macro-levels.

**Supplementary Information:**

The online version of this article (10.1007/s11136-021-02766-9) contains supplementary material, which is available to authorized users.

## Introduction/background

Patient-reported outcomes (PROs), and by extension patient-reported outcome measures (PROMs), are increasingly used to inform healthcare decision-making. The decisions that PROs inform can be considered at the level of patient care (micro), the healthcare organization (meso), and health policy (macro). There is ample evidence that response shift can affect PROs [1, 2]. Response shift is defined as a change in the meaning of one’s self-evaluation as a result of changes in internal standards (recalibration), values (reprioritization), and/or conceptualization of the target construct (reconceptualization) [3], which may result in measurements at two or more time points not being comparable. Evolving perspectives of measurement validity place increasing emphasis on the inferences, judgements, and decisions based on measurement scores [4]. It is therefore important to consider the potential implications of response shift when measures of change in PROs over time are used to inform decisions. For example, when we assess patients’ perceived health status at two time points, we can take their responses to that item at each time point at face value, even if response shift has occurred in the interim. However, if we want to measure *change* in perceived health status over time, inferences we draw from the observed change in that item may be invalid, leading to ill-informed decisions. The possibility of response shift must therefore be considered when measuring changes in PROM scores over time and when comparing differences in PROM scores between people (e.g., when comparing people who experienced response shifts with those who did not).

To date, the implications of response shift for healthcare decision-making have rarely been investigated [5]. Our goal is to provide a framework regarding the impacts of response shift at micro-, meso- and macro-levels of healthcare decision-making. In so doing, we intend to raise awareness and provide guidance for PRO researchers who play an important role in informing healthcare providers, healthcare leaders, health policymakers, and regulators regarding the implications of response shift. This work is part of an international, interdisciplinary collaboration (see Appendix for the participating members) to synthesize the research on response shift to date [6–8].

## Analytic frame

Our analysis is informed by perspectives of measurement validity, hermeneutic philosophy, and healthcare decision-making. From a measurement validity point of view, the impact of response shift on healthcare decision-making ultimately pertains to whether the inferences we make are valid, and subsequent actions and decisions made on PROs are well founded. This view of measurement validity is based on the foundational work by Messick [4, 9] and, subsequently, Zumbo [10–13], which has received increasing attention in PRO measurement [14–16]. Response shift threatens the measurement assumption that the process and frame of reference by which a person responds to PROM items are consistent over time [17]. In other words, response shift occurs when observed scores (i.e., responses to PROM items) do not reflect the construct of interest in the same way at different points in time [18, 19]. As a consequence, the meaning of the PROM score will not be consistent over time. Our inferences, actions, and decisions made on PROs must therefore take into account the possibility that PROM scores may be influenced by response shift.

Building on the theoretical premises of measurement validity, we take a hermeneutic philosophical lens to gain further insight into how the meaning of PROM scores may change over time [20] and the consequent implications for micro-, meso-, and macro-level healthcare decision-making. At a foundational level, the use of PROs involves an interpretive process of understanding the meaning of individuals’ responses to PROM items. We specifically drew upon Gadamerian hermeneutics [21, 22] because it draws attention to the dialectical processes (a.k.a. hermeneutic circle) that provide insights into how different stakeholders interpret and use PROM results. Dialectical processes refer to the interplay between patients’ prior ideas and experiences and the assumptions held by researchers and clinicians about health. This interplay constitutes a dialectical process of navigating differences in how patients interpret and respond to questions about their health, and how researchers and clinicians interpret the results. We accordingly structure the micro-, meso-, and macro-level implications of response shift based on the following three tenets from Gadamerian hermeneutics: (1) the use of PROMs involves the interpretation of contextual elements; (2) interpretation of PROM results is an ongoing dialectical interaction; and (3) the integration of PROM data in decision-making requires openness and reflexivity. We formulated corresponding key guiding questions to structure our analysis in an effort to critically analyze the implications of using PROMs for healthcare decision-making (see Table 1).

**Table 1:** Tenets and guiding questions for considering response shift implications based on hermeneutics

Tenet	Guiding questions
Tenet 1. The use of PROMs involves the interpretation of contextual elements	What is the purpose of using PROMs in this context?
	Is the context specific to an intervention, program or healthcare model?
Tenet 2. Interpretation of PROM data is an ongoing dialectical interaction	How are assumptions or beliefs influencing the interpretation and use of PROM results?
	Is it assumed that an intervention would improve PROM scores?
Tenet 3. The integration of PROM data in decision-making involves ideally openness and reflexivity	Can response shift affect the interpretation of PROM results?
	If response shift is identified, how does this affect the inferences about the meaning of PROM scores?

**Fig. 1:**
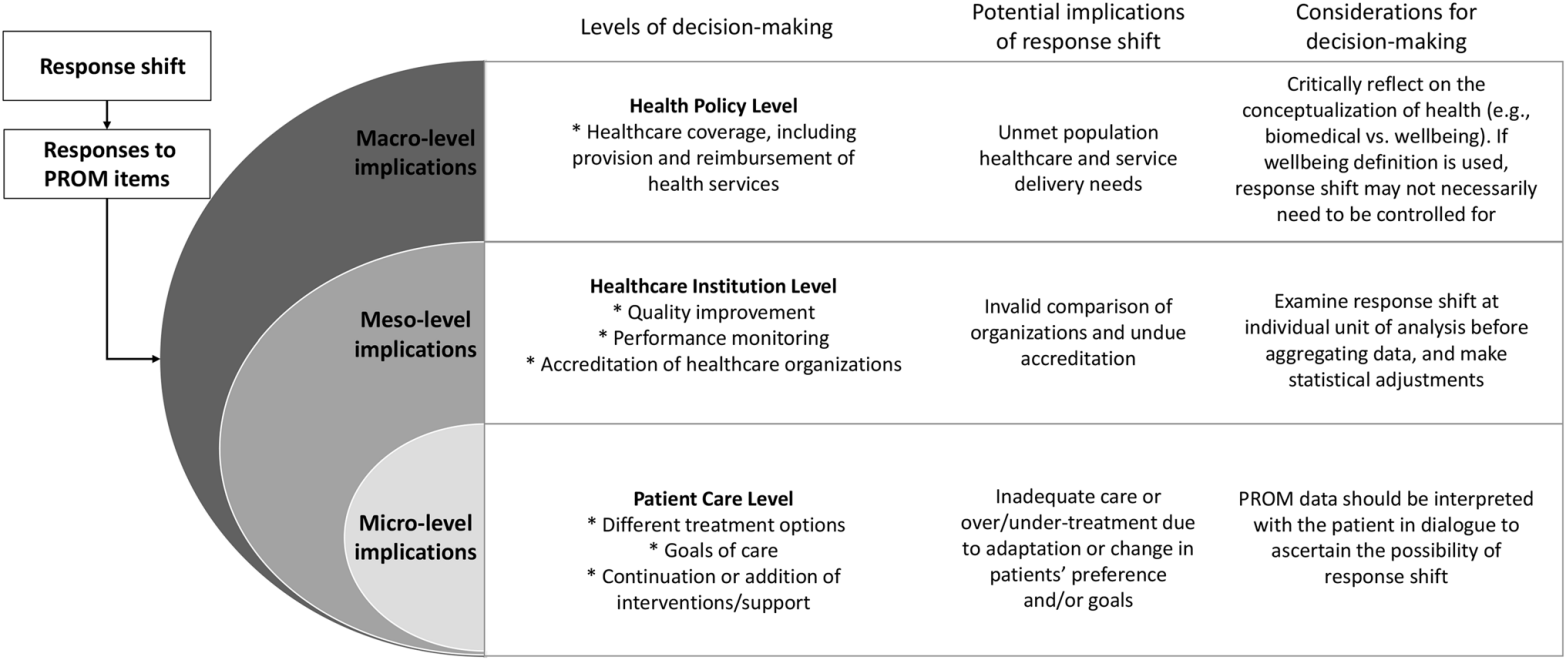
Potential response shift implications for use of PROM data at micro-, meso-, and macro-levels of healthcare decision-making

Additionally, our analysis is informed by a recognition that there are many different types of healthcare decisions that need to be made for a range of different purposes. We therefore adopted a healthcare system’s orientation towards micro-, meso-, and macro-levels of decision-making that draws attention to the different priorities and perspectives of stakeholders [23, 24]. The micro-level focuses on the use of PROM data in clinical practice for the purposes of informing clinical decisions about a patient’s care. This is particularly pertinent in shared decision-making where clinicians and patients share information about PROs and the best available evidence to make decisions about treatments, goals of care, and continuation or addition of interventions or supportive services, in relation to patients’ preferences [25]. At the meso-level, PROM data are used by healthcare managers and leaders for quality improvement, performance monitoring, and accreditation of different healthcare services and organizations. The macro-level focuses on the overall healthcare system where PROM data are used by government leaders and decision-makers to inform health policy regarding healthcare coverage, including the provision and reimbursement of healthcare services. The levels are interrelated, where the macro-level informs and is informed by impacts at the meso- and micro-levels, and the meso-level informs and is informed by impacts at the micro-level. Figure 1 provides an overview of the different types of decisions made at each level and the concurrent interdependent relationships between these levels. By organizing our examination into micro-, meso-, and macro-levels of decision-making, we also provide a framework for developing recommendations for mitigating potential unintended consequences of response shift at each level.

## When does response shift occur?

Response shifts can result from any event or experience that causes a person to think differently about internal standards, values, or conceptualization of their health. Response shifts can occur as a result of sudden *acute conditions*, such as cerebral hemorrhage, head injury, and spinal cord injury [26–28]. Response shifts can also result from experiences in living with potentially progressive *chronic conditions*, including multiple sclerosis, cancer, diabetes, HIV/AIDS, chronic obstructive pulmonary disease, and sleep apnea [1, 29–33]. Chronically ill patients with stable health are less likely to experience response shifts [34]. Response shifts have also been demonstrated in patients whose health improved, for example in patients with pain undergoing total knee arthroplasty, hearing impaired adults receiving hearing aid fitting, and edentulous patients receiving denture treatment [35–37]. *Healthcare interventions* could also lead to response shifts [38]. Some interventions are designed to promote reframing how one thinks about one’s health and are particularly likely to produce response shifts, in fact, as a desired outcome [39, 40]. For other interventions, response shifts may be an unintended consequence (e.g., invasive surgery or toxic cancer treatment [41]). Finally, response shifts could occur as a result of *life events* that are not defined by specific diseases or chronic conditions (e.g., experiences associated with human development and aging) [42, 43]. Another example includes informal caregivers who may experience response shifts as a result of the challenges and experiences associated with caregiving [44]. In all of the above situations, it is important to consider that some people may be more susceptible to response shifts than others.

While various methods exist for evaluating the occurrence and magnitude of response, the “then-test” has been used most frequently (see Sébille et al. for explanation of methods and limitations [8]). The “then-test” entails a retrospective assessment administered at follow-up asking for a re-evaluation of one’s functioning at the time of the first assessment (pre-test) [17]. However, although this test explicitly operationalizes the notion of response shift, it is also sensitive to recall bias. A meta-analysis by Schwartz et al. (conducted in 2006) of 19 studies using the “then-test” indicated that the magnitudes of response shift effects are generally small and vary by the type of health outcome being measured (e.g., symptoms versus physical function) [45]. In fact, Rapkin and Schwartz have linked response shift to a particular type of self-reported outcome, i.e., evaluation-based outcomes, as opposed to performance-based or perception-based outcomes [46]. Nonetheless, it should be noted that even a small response shift effect may change conclusions [41].

It is also important to consider unanticipated response shift effects that are often overlooked, which can be exemplified for four basic research designs (Table 2). In cross-sectional observational designs, response shifts may have taken place prior to study entry. For example, differential exposure to factors leading to response shifts (e.g., length of time living with a chronic condition) could result in differences between respondents in their frames of reference for interpreting the PROM items and response scales. In longitudinal interventional studies with or without cost-effectiveness analyses, treatments that are compared may induce response shifts in different outcomes, or with different magnitudes, and/or directions of response shift. In these instances, erroneous conclusions may be drawn regarding the differences in PROM results between groups, changes in PROM results over time, and the preference for and cost-effectiveness of one treatment over another.

**Table 2:** Implications of response shift for study results

Research design	Occurrence of response shift	Effect on results	Example
**Cross-sectional observational:** comparing groups at the same time point	Prior response shifts could result in different standards, values, or interpretations (meaning) of PROs between groups. For example, events in the past may have induced changed perspectives (earlier response shifts), resulting in either higher or lower levels of PROM results than would be expected based on health status.	Response shifts that differ between groups may affect conclusions about differences in PROM results, e.g., insignificant (mitigated) differences where differences exist or significant (amplified) differences where none exist.	Breast cancer patients were found to report comparable or superior PROM results (including anxiety and depression) in comparison with healthy women [90].
**Longitudinal observational:** assessing one group over time	Health *deterioration* leading to lowered standards, changed priorities, and conceptualizations. Health *improvement* leading to raised standards, changed priorities, and conceptualizations.	This type of response shift may result in higher levels of QoL than would be expected based on health status. Such response shift may result in lower levels of PROM results than would be expected based on health status.	Assessing the impact of chemotherapy and/or radiotherapy on cancer patients’ QoL: Adaptation to the toxic side effects may induce response shifts that obfuscate the negative impact of these side effects on QoL [91]. Assessing the impact of knee arthroplasty on QoL: Adaptation to the health state improvement, being able to walk again, might evoke response shifts that obfuscate the improvements in QoL [36].
**Longitudinal interventional**	Treatment may induce response shifts when patients need to adapt to the changing health state. For example, treatments that are compared may induce response shifts in different outcomes, or magnitude and/ or direction in the same outcomes.	The comparison of outcomes that are differentially affected between groups by response shifts may lead to erroneous conclusions, e.g., insignificant (mitigated) differences where differences exist or significant (amplified) differences where none exist, also when randomization has taken place.	Patients with myelofibrosis treated with ruxolitinib reported improvement in functional domains and global QoL whereas patients receiving placebo-reported deterioration [92]. Longer follow-up indicated a small decline in QoL in the treatment condition; the placebo condition was not followed up. Whereas not measured, authors hypothesized that the decline might be related to response shift [93]. The difference between treatment and placebo conditions might have been amplified by the occurrence of response shift, an effect that may have decreased over time due to adaptation.
**Longitudinal interventional, including cost-effective analysis**	Same as longitudinal interventional.	Differences in response shifts between two treatments may lead to incorrect cost-effective analyses and an incorrect preference for one treatment over another, also when randomization has taken place.	Comparing conventional (invasive) cardiac valve surgery and a percutaneous noninvasive valve implantation: Surgery may induce more response shift, leading to improved PROM scores that would be expected based on health state. Since surgery is generally cheaper than a valve implantation, the combination of improved PROM scores and lower costs may lead to an incorrect preference for surgery over a percutaneous intervention (hypothetical example in [5]

## Implications of response shift at different levels of healthcare decision-making

### Micro-level decision-making

At the micro-level, PROM data are used to inform decisions about different treatment options, goals of care, and the need for continuation or addition of interventions or supportive services (see Fig. 1 and Table 3). These types of decisions may be informed by PROMs data from individual patients [47, 48] as well as aggregate PROMs data about groups or populations [49].

**Table 3:** Examples and potential implications of response shift at the individual patient (micro)-level based on Gadamerian hermeneutics

Type of decision	Response shift example	Contextual elements: purposes for using PROMs	Dialectical processes: assumptions influencing interpretation	Openness and reflexivity: impact of response shift on inferences about meaning of the scores	Potential consequences: How response shift affects decision-making
Choice between different treatment options	Patients with temporal lobe epilepsy participating in a surgical randomized trial were found to reprioritize the importance of domains constituting QoL [50].	The Quality of Life in Epilepsy Inventory-31 (QOLIE-31), a 31-item questionnaire that comprised of 7 domains (overall quality of life, social function, fatigue, emotional well-being, seizure worry, memory, and medication effect) was used to assess surgical and medical treatment.	The traditional assumption in evaluating treatment effectiveness in individuals with epilepsy is that freedom from seizures and number of antiepileptic drugs prescribed continues to be the highest priority before and after surgery.	Over time, patients with epilepsy placed less importance on seizure worry and more on social function items.	If response shift (reprioritization) is ignored, patients may be prescribed interventions (e.g., drugs) that could have adverse effect on patient’s social function, which may be as, or even more, important than seizure management.
Decision about goals of care	Patients at 6- and 24-weeks post-stroke who were discharged home from an acute hospital admission were found to experience reconceptualization and reprioritization response shifts [29].	The Patient-Generated Index (PGI) was used. This individual-level measure asks patients to identify five important life domains and weigh each domain according to its importance. Patients were additionally interviewed post-stroke to assess whether they indicated to have experienced response shift.	Persons with stroke experience a sudden loss of function, which gradually recovers with time. The implicit assumption of this study was that at different stages of recovery, patients may find different life domains important (reconceptualization) and/or how change how important different domains are relative to one another (reprioritization).	Of the 46 interviewed patients, 13 (28%) expressed verbalizations reflecting a response shift. The majority selected entirely different life domains at 24 weeks and changed their importance accordingly.	Response shift may occur during recovery (rehabilitation intervention and natural recovery). When ignored, this could lead to inappropriate goal setting that is not aligned with the domains of importance to the patient nor with his/her priorities of that moment.
Decisions about the need for continuation or addition of interventions or supportive services	In patients diagnosed with major depressive disorder participating in a randomized trial comparing psychotherapy with anti-depressant medication, response shift was found, with this response shift effect being larger in the psychotherapy group [39].	The Beck Depression Inventory (BDI), a 21-item questionnaire designed to measure behavioral symptoms of depression, was used.	The implicit assumption of this study is that psychotherapy includes education about depression, which could change patients’ concept of the disorder and the way they view their symptoms, and in turn influence the way in which patients respond to PROM items.	Patients who received psychotherapy, compared to the medication group, were better at assessing their level of symptomology, viewed depression as a more unified concept, and had become more aware of their depressive symptoms.	Patients may not receive interventions/services such as psychotherapy because clinicians may believe that the worsened PROM scores were the result of the intervention rather than response shift, which allowed patients to be more aware of their condition and its symptoms.

When a choice needs to be made between *different treatment options*, the patient’s health status, preferences, and values need to be taken into account, in a process of shared decision-making, if the patient so desires. It is possible that the relative importance that patients place on different PRO domains changes over time (i.e., reprioritization response shift). For example, response shift analyses of the Quality of Life in Epilepsy Inventory-31 (QOLIE-31) comparing surgical and medical treatments for epilepsy suggest that patients with epilepsy who receive surgical treatment place relatively less importance on “seizure worry” and more on the social function domain over the course of time in their illness trajectory in relation to the two treatments [50] (see Table 3). These results suggest that social function is an important consideration when deciding between surgical and medical treatments. From a hermeneutic point of view, consideration of “dialectical interaction” can help to further explore the possibility of a shift in relative importance by reaching a shared understanding between the patient and the clinician in the context of shared decision-making. For example, if this response shift is ignored, clinicians might erroneously focus on seizure management by prescribing drugs that adversely affect patients’ social function, which has now been identified as being more important. Aggregated PROM data may inform decisions between different treatment options based on published results of intervention studies or patient registries that used PROMs with comparable patients undergoing the target intervention. As PROM results have been shown to be predictive of other outcomes such as re-operations [51], re-occurrence of index events [52], and mortality [53, 54], their use for individual decision-making is likely to increase. A particular challenge regarding the use of such aggregated PROM data is that response shift may have occurred but not taken into account (e.g., by statistically adjusting for the occurrence of response shift), thereby potentially misinforming individual decision-making for patients who are likely to undergo response shift.

When decisions need to be made about the *goals of care,* it is important to realize that such goals may change over time, depending on the course of the disease trajectory and the preferences of the patient. At different stages, patients may find different life domains relevant (reconceptualization) and prioritize those differently (reprioritization). For example, Ahmed et al. [29] found that the relative importance of different health domains in the Patient-Generated Index (PGI) changed at different stages of recovery following a stroke. When such response shifts are ignored, inappropriate goal setting that is not aligned with the patient’s current priorities may occur (Table 3). To prevent inappropriate goal setting, hermeneutics can be used to ask open and reflexive questions with the patient such as: How has the patient’s thinking changed, and what would the patient do differently now? In addition, if a condition cannot be cured (i.e., most chronic conditions), patients can be taught to change their values and frame of reference as part of a process of adaptation. In those situations, response shift itself is the goal of treatment, as is frequently the case in rehabilitation [38, 55, 56], patient education (see, for example, Kiresuk and Sherman [57], Reuben and Jennings [58], and Nolte et al.[59]), and most psychological treatments (e.g., cognitive behavioral therapy) [60].

When a decision needs to be made about *continuation or addition of interventions or supportive services,* patients’ self-reports on their health status can be used to assess the changes they may have experienced. Routine collection of PROM data in clinical practice has been recommended for purposes of monitoring the impact of health conditions and interventions from the patient’s point of view. Examples include the increasing use of PROM data in electronic medical records of healthcare providers, as well as personal health records that are maintained by patients themselves [61]. The PROM data can be used to determine whether continuation or addition of interventions or supportive services are required to reduce symptoms or better address functional problems or concerns of the patient. A particular risk with this type of use of PROM data is that ignoring response shifts can lead to erroneous conclusions about whether interventions and supportive services are achieving the desired outcomes. For example, education about depression is often provided as part of psychotherapy treatment aimed at changing a patient’s perspective on depression and how they view depressive symptoms. A study by Fokkema et al. [39] showed that the resulting reconceptualization response shift could result in deterioration, rather than improvement in PROM depression scores, due to increased awareness and recognition of their depressive symptoms (see also Table 3). Subsequently, a clinician may attribute this deterioration to the treatment itself (e.g., education with psychotherapy) and may therefore no longer continue the treatment even though the patient has experienced an improvement in depressive symptoms. This situation illustrates the importance of interpreting PROM depression scores in dialogue with the patient as the basis for making an accurate assessment of the change of the patients´ health status over time.

### Meso-level of decision-making

At the meso-level, the focus is on the use of PROM data for decisions made by healthcare managers and administrators for quality improvement, performance monitoring, reimbursement, and accreditation [62, 63] (see Fig. 1 and Table 4). Meso-level decision-making uses aggregated PROM data. Translating individual-level data to group-level decision-making could lead to biases and skewed inferences for multiple reasons, including failure to account for individual-level response shifts.

**Table 4:** Examples and potential implications of response shift at the healthcare institution (meso) level based on Gadamerian hermeneutics

Type of decision	Response shift example	Contextual elements: purposes for using PROMs	Dialectical interaction: assumptions influencing interpretation	Openness and reflexivity: impact of response shift on inferences about meaning of the scores	Potential consequences: How response shift affects decision-making
Decision about quality improvement initiatives	The arthritis foundation assessed their chronic disease self-management program and found that participants responded differently to questions posed after the program than they did to the same questions posed before [40].	The Health Education Impact Questionnaire (HEI-Q) was used to assess response shift in measuring the benefits of self-management programs.	Traditional evaluation of health intervention programs assumes that participants do not change the way they perceive their health over the period of the program.	The majority of participants (~ 87%) had an altered internal standard of measurement on at least 1 item, which may obscure self-management programs as having small or no effects.	Quality improvement initiatives of self-management programs may not show any difference unless response shift is used as an outcome or at least is taken into account.
Decisions based on performance monitoring	In a study comparing primary healthcare organizational models and PROs, PROM scores over a period of 18 months of patients in various clinics were equivalent despite differences in complex health problems [65].	The Medical Outcomes Short Form-36 (SF-36), the Health Assessment Questionnaire (HAQ), the Minnesota Living with Heart Failure Questionnaire, the Chronic Respiratory Questionnaire, and the Audit of Diabetes-Dependent Quality of Life (ADDQoL) were used to assess primary healthcare organizational models.	The implicit assumption of the study is that certain organizational models (e.g., community-oriented model) may be more effective in improving health outcomes in patients with chronic disease.	The largest portion of the variance in PROM scores was attributed to individual factors (e.g., number of comorbidities), whereas only 4.3% was accounted for by organizational factors. There was not much change in health status and QoL over the study period, which the authors attributed to the slow progress of the conditions, attrition of the worst cases, and the possible occurrence of response shift as a result of adaption to limitations.	Ignoring response shift could lead to PROM scores of community practices being equivalent to other types of primary healthcare organizations, even when community practices have more patients with complex health needs
Accreditation of healthcare organizations	Residents participating in an adolescent medicine rotation training program aimed at improving their ability to manage adolescents’ health issues demonstrated a significant increase in self-perceived skill levels for all assessed domains. Participation in didactic instruction did not yield significant additional benefit for any of the assessed domains. The program was required by Accreditation Council for Graduate Medical Education [67].	Survey-based resident self-assessment was used to compare differential gain in self-assessed skills and confidence in residents exposed to a didactic curriculum to those who participated in the clinical rotation-only group. Assessments took place after the program and were administered as a post- and then-test.	Post-then comparison was assumed to reduce response shift bias seen in traditional pre- and post-test design particularly with self-assessment tools.	There were no differences in self-assessed skills and confidence in residents exposed to a didactic curriculum to those who participated in the clinical rotation-only group as the post-then comparisons took possible response shift effects into account.	If the possibility of response shift is ignored, groups that should differ may have comparable PROM scores (conversely, differences could be amplified where none exist), which could result in undue accreditation.

Decisions about *quality improvement* initiatives involve patients’ evaluations of healthcare systems and use of PROM data. Often these efforts are aimed at improving efficiency of healthcare delivery, to optimize limited resources and control costs for the health system. These within-system efforts are usually directed and used by administration. Response shift is among the threats to valid inferences about impacts of such initiatives, but is rarely examined. For example, a study by Osborne et al. [40] reveals that participants of a self-management program may respond differently to evaluative questions about their health posed after the introduction of the program compared to before. Results suggest that 87% of the participants experienced response shift (see Table 4). For example, response shift occurred when participants realized that their health before the program was worse than they thought. This would result in an underestimation of the improvements in PROM scores over time. If the possibility of response shift is ignored, this could lead to an ill-informed meso-level decision to cancel or alter the self-management program. To prevent the possibility of this ill-informed decision, hermeneutics can be used to ask participants about contextual factors that could have altered their perspectives and to seek a balanced appreciation of the diversity of perspectives when meso-level decisions are being made based on PROMs results.

Other meso-level decisions relate to *performance monitoring* [64], which involves between-system comparisons based on PROM data. For example, the Consumer Assessment of Healthcare Providers and Systems (CAHPS) survey is administered by the Centers for Medicaid and Medicare (CMS) in the US as part of their public reporting or reimbursement programs. The survey provides information on the quality of health services at multiple levels of the healthcare system and allows comparisons across healthcare providers. Patient-reported “experience” variables are an important component of this comparison, and randomly selected hospital patients are surveyed cross-sectionally or longitudinally. However, comparisons between systems may potentially provide misleading results if response shift is ignored. For example, a study by Feldman et al. [65] comparing different primary healthcare organizational models found that PROM scores were equivalent despite differences in complex health problems presented in those organizations (for a similar attempt of large scale PROM use in the UK, see [66]). A Hermeneutic perspective of openness and reflexivity points to the importance of exploring the possibility of response shift in patients with complex problems. This may, for example, occur as a result of patients adapting to their limitations or living with comorbidities for a prolonged length of time.

Similarly, a*ccreditation of healthcare organizations* that relies on evaluation-based outcomes may be impacted by response shift. In addition to PROs, accreditation also often relies on other evaluation-based outcomes (e.g., self-perceived competence or skill) in different populations (e.g., healthcare professionals). For example, Ruedinger et al. [67] chose a different approach in an accreditation study for the adolescent medicine training program. They compared a didactic curriculum intervention group with a clinical rotation-only group at one point in time following these interventions. They administered a post- and then-test to examine retrospectively the change pediatric resident trainees may have undergone. While recognizing other types of biases present (e.g., recall bias and social desirability bias), the authors noted that this study design helped to reduce response shift bias and allowed for comparable self-assessment scores. With many organizations (e.g., National Quality Forum) supporting increased collection of PROM data and evaluation-based outcomes of other populations (e.g., healthcare professionals) for quality measurement [68], questions regarding how to best account for response shift in quality rating, reimbursement, and accreditation decisions are likely to grow.

### Macro-level of decision-making

At the macro-level, implications of response shift focus on the use of PROMs by governments for health policy purposes [69, 70] (See Fig. 1, and Table 5). The effect of response shift on PROM results readily translates from the micro- and meso-level to the macro-level decisions regarding healthcare coverage. For example, a study of the September 11 terror attacks illustrates how a population-level calamity may result in response shifts resulting in reprioritization of relationships, compassion, and spirituality in a large segment of the population [71]. Consequently, when ignoring response shift, decisions about healthcare coverage may not fully address the need for services corresponding with these changing priorities. Decisions about healthcare coverage, including *provision and reimbursement of healthcare,* are increasingly informed by PROM data. Health technology assessment supports such decision-making by synthesizing and evaluating diverse types of evidence, typically by aggregation of individual responses to patient preference measures [72]. In addition to evidence of effectiveness, healthcare needs and cost-effectiveness are important criteria for provision and reimbursement of care. Whereas treatment effectiveness (e.g., as investigated with randomized clinical trials) and healthcare needs (see micro-level) are typically assessed in patients, there is debate about whether values in cost-effectiveness analyses should preferably be provided by patients or by the “healthy population” or public. The possible occurrence of response shift in patients adds importance to this debate, as the public may not be representative for patient populations. Quality-adjusted life years (QALYs) are often used as an outcome measure in cost-effective analyses that provides a uniform economic reference framework across healthcare. QALYs are derived from patients’ responses to PROMs by combining length of life with quality of life (QoL). As such, QALYs have an evaluative component that is particularly susceptible to response shift. Here, quality is measured by utility, which reflects the value of QoL on a scale anchored at 100% for perfect health and 0% for health that is as bad as dead. Economically, larger QALY gains justify higher costs. Guidelines in many countries prefer utilities to be assessed from a societal perspective [71, 73, 74]. As taxes and health insurance premiums are mostly paid by the general public, they should also have a say in what is important. This societal approach has the additional advantage that it reduces the impact of response shift. If patients adapt to a particular condition and no longer experience the burden, then relieving that condition would not represent a QALY gain, thus reducing the extent to which costs are acceptable. The general public mostly does not experience the particular condition, so they cannot adapt to it either. As a result, the public generally gives more weight to conditions than patients undergoing the condition, with the exception of depression [75].

**Table 5:** Examples and potential implications of response shift at the health policy (macro) level based on Gadamerian hermeneutics

Type of decision	Response shift example	Contextual elements: purposes for using PROMs	Dialectical interaction: assumptions influencing interpretation	Openness and reflexivity: impact of response shift on inferences about meaning of the scores	Potential consequences: How response shift affects decision-making
Healthcare coverage	Following the September 11 terror attacks, Manhattan residents were assessed for post-traumatic stress disorder. While most residents reported one or more stress symptoms, positive effects were also reported, including closer relationships with others, and increased compassion and spirituality [71].	Various measures were used to assess the psychological processes mitigating the trauma of September 11, including: the 20-item Center for Epidemiological Studies-Depression Scale, the eight-item Hope Scale, and the 20-item Spiritual Meaning-Long Form.	Response shift has generally been assumed to be an individual-level phenomenon, triggered by a perceived change in an individual’s health status as a result of treatment or disease, rather than a population-level phenomenon.	The event triggered a reprioritization of values with close relationships, compassion, and spirituality leading respondents to perceive PROM items independent of any actual change in functional status.	If population response shift is ignored, large segments of the population may face unmet needs (e.g., spiritual care) that are not covered by healthcare or addressed in the current treatment guidelines.
Provision and reimbursement of healthcare services	The objective of this study was to describe a framework for understanding the potential mechanisms that play a role in health state valuations when making reimbursement decisions [94].	Health state utilities (e.g., quality-adjusted life years) are used for various healthcare decisions, including reimbursement of treatments based on their cost-utility.	Health state utility measure is assumed to be relatively similar in outcomes for comparative cost/utility analyses between the general public and patients.	The general public who are asked to imagine experiencing health states assign lower utilities to those states than do patients who are actually experiencing these states.	If differences in values provided by respondent groups (e.g., public vs. patients) are ignored such that patients with serious disabilities report high utility scores due to response shift, funding for treatment may be withdrawn based on inaccurate assessment of utility.

Since societal valuations still need to be linked to measurements from patients who are experiencing the conditions and treatments, utility scores may be attached to disease-specific questionnaires. This may be a relatively objective and sensitive approach, but tends to overestimate utility in poor health states [76]. Instead, generic questionnaires have been developed that span a wide range of health and QoL domains, and for which societal valuations are available to calculate utility scores [77–80]. These questionnaires may reduce the impact of response shift by asking patients to provide a more factual *description* of their health, whereas the more subjective *valuation* is provided by the non-adapting general public. Over the past decades, utility scores have been estimated in many countries, acknowledging that calibration, prioritization, and conceptualization may differ over place and time [81]. As utility questionnaires have been criticized for favoring the cure sector, current research includes attempts to capture reprioritization and reconceptualization for the care sector and in particular end-of-life care [82, 83].

### Considerations for decision-making

Two key questions need to be considered for healthcare decision-making at the micro-, meso-, and macro-level. First, should decision-making be based on adjusted data (where response shift is taken into account), or unadjusted data (where response shift affects the scores)? Second, will the decision-making be informed by retrospective data that may be affected by response shift, or by the possible occurrence of response shift in the future, using prospective data? The answers to these questions will depend on the particular decision at stake, the specific patient, organization, or population under consideration, and the nature and type of the health definition used. Additional considerations pertaining to each level of decision-making also need to be taken into account.

First, at the micro-level, healthcare providers are advised to interpret a patient’s self-reported outcomes data in dialogue with the patient [84] to ascertain the possibility of response shift, and let this inform the decision that is to be made; otherwise, a simplistic routine use of PROMs may do more harm than good. However, when dialogue with the patient is not possible, secondary sources of information could also be considered in determining whether adaptation, or other factors influencing response shift, may be taking place. For example, when patients have been exposed to interventions focused on facilitating an adaptive process (e.g., developing better coping skills after chemotherapy), an improvement in PROM scores may be interpreted as evidence of adaptation irrespective of there being any change in a person’s actual health status [55, 85].

Second, at the meso-level, it is important to consider that decision-making is often based on cross-sectional data, or on system-level longitudinal data, in which within-subject (individual-level) longitudinal change is not explicitly measured. In these cases, response shift may not be identified even if it occurred. Where possible, we therefore recommend obtaining and analyzing longitudinal individual-level data to examine implications of response shift, and making statistical adjustments to account for the possibility of differential response shifts in some groups of people (e.g., by using latent variable methods [86], while taking limitations of current approaches into account [8]). Aggregating these adjusted PROM data for the purposes of quality improvement, performance monitoring, or accreditation will provide a sound basis for meso-level decision-making.

Third, at the macro-level, it is particularly important to critically reflect on the conceptualization of health and its implications for healthcare policy. For example, if the biomedical definition of health as the absence of pathology is used [87], then response shift must be controlled for to distinguish a change in individuals’ reported health status from their biomedical health status. In contrast, if a broader and positively phrased conception of health is used to include “physical, mental, and social well-being” [88] and the “ability to adapt and self manage” [89], then response shift may not necessarily need to be controlled for.

## Epilogue

The key message of this paper is that response shifts could either reduce or increase the size of the change in PROM results among people with the same health state, thereby providing misleading information, which in turn may affect the quality of healthcare decision-making at the micro-, meso-, and macro-levels. If response shift is measured and taken into account [86], the results are expected to be more sensitive by teasing out the effect of adaptation leading to response shift from the actual health change, thereby providing a sound basis for medical decision-making. This is one of the first papers that started to outline possible influences of response shift on healthcare decision-making [5]. The currently described impacts may not be exhaustive nor sufficiently nuanced. Given the novelty of considering response shift in the context of healthcare decision-making, we need empirical studies to examine under what circumstances response shift affects which types of decisions to what extent. Such studies may also teach us how response shift effects relate to other known biases due to, for example, other forms of response bias, representativeness, and missing data. With more empirical data available, we also expect to improve our understanding of how to account for response shift to obtain a more stringent basis for decision-making. It is hoped that increased awareness of the potential, heretofore neglected influence of response shift, will improve the quality of healthcare decision-making at all levels.

## Supplementary Information

Below is the link to the electronic supplementary material.

(PDF 419 kb)
