# Supplementary material for: Implications of response shift for micro-, meso-, and macro-level healthcare decision-making using results of patient-reported outcome measures
Source: Qual Life Res. 2021 Mar 2;30(12):3343–57. doi: 10.1007/s11136-021-02766-9 (PMC8602130; doi:10.1007/s11136-021-02766-9)
Supplement: Supplementary file 1 — (PDF 419 kb) [file 11136_2021_2766_MOESM1_ESM.pdf]

Appendix: Members of the Response Shift –in Sync Working Group

- Amelie Anota, Department of Human and Social Sciences INSERM UMR 1098, Cancer Care Center Léon Bérard, Lyon, France;
- Oluwagbohunmi Awosoga, Faculty of Health Sciences, University of Lethbridge, Lethbridge, Canada;
- Olawale F. Ayilara, Department of Community Health Sciences, University of Manitoba, Winnipeg, Canada;
- Ruth Barclay, Department of Physical Therapy, University of Manitoba, Winnipeg, Canada;
- Jan R. Böhnke, University of Dundee, School of Health Sciences, Dundee, United Kingdom;
- Anita Brobbey, Department of Community Health Sciences, University of Calgary, Calgary, Canada;
- Cynthia Chauhan, Patient Representative, Wichita, KS, USA;
- Lori Frank, Behavioral & Policy Sciences, RAND Corporation, Arlington, VA, USA;
- Bernice G. Gulek, University of Washington, Harborview Medical Center, Seattle, WA, USA and Washington State University, College of Nursing, Spokane, WA, USA;
- Wilbert van den Hout, Medical Decision Making, Department of Biomedical Data Sciences, Leiden University Medical Center, Leiden, The Netherlands;
- Cecile J. W. Janssens, Department of Epidemiology, Rollins School of Public Health, Emory University, Atlanta, GA, USA;
- Lene Kongsgaard Nielsen, Department of Haematology, Quality of Life Research Center, Odense University Hospital, Odense, Denmark and Department of Internal Medicine and Cardiology, Regional Hospital Viborg, Viborg, Denmark;
- Jae-Yung Kwon, School of Nursing, University of British Columbia, Vancouver, Canada;
- Oluwaseyi Lawal, Department of Community Health Sciences, University of Calgary, Calgary, Canada;
- Lisa M. Lix, Department of Community Health Sciences, University of Manitoba, Winnipeg, Canada;
- Nancy Mayo, Department of Medicine, Division of Clinical Epidemiology, Center for Outcomes Research and Evaluation (CORE), McGill University, Montreal, Canada;
- Leah McClimans, Department of Philosophy, University of South Carolina, Columbia, SC, USA;
- Sandra Nolte, ICON GmbH, Munich, Germany and Charité – Universitätsmedizin Berlin, corporate member of Freie Universität Berlin, Humboldt-Universität zu Berlin, and Berlin Institute of Health, Medical Department, Division of Psychosomatic Medicine, Berlin, Germany;
- Frans J. Oort, Research Institute of Child Development and Education, University of Amsterdam, Amsterdam, The Netherlands;
- Nikki Ow, School of Physical and Occupational Therapy, Center for Outcomes Research and Evaluation (CORE), McGill University Montreal, Canada;
- Tolulope T. Sajobi, Department of Community Health Sciences and O'Brien Institute for Public Health, University of Calgary, Calgary, Alberta, Canada;
- Richard Sawatzky, School of Nursing, Trinity Western University, Langley, Canada;
- Véronique Sébille, UMR INSERM 1246, SPHERE "methodS in patient-centered outcomes and HEalth ResEarch", University of Nantes, University of Tours, Nantes, France;
- Mirjam A. G. Sprangers, Department of Medical Psychology, Amsterdam University Medical Centers, Research Institute Amsterdam Public Health, Amsterdam, The Netherlands;

- Antoine Vanier, UMR INSERM 1246, SPHERE "methodS in patient-centered outcomes and HEalth ResEarch", University of Nantes, University of Tours, Nantes, France;
- Mathilde G. E. Verdam, Department of Methodology and Statistics, Institute of Psychology, Leiden University, Leiden, The Netherlands.
